# Can Melatonin Help Us in Radiation Oncology Treatments?

**DOI:** 10.1155/2014/578137

**Published:** 2014-05-11

**Authors:** Ehsan Mihandoost, Alireza Shirazi, Seied Rabie Mahdavi, Akbar Aliasgharzadeh

**Affiliations:** ^1^Department of Medical Radiation Engineering, Tehran Science and Research Branch, Islamic Azad University, Tehran, Iran; ^2^Department of Medical Physics and Biomedical Engineering, Faculty of Medicine, Tehran University of Medical Sciences, Keshavarz Boulevard, Poursina Avenue, Tehran, Iran; ^3^Department of Medical Physics, Faculty of Medicine, Iran University of Medical Sciences, Tehran, Iran; ^4^Department of Radiology and Medical Physics, Faculty of Paramedicine, Kashan University of Medical Sciences, Kashan, Iran

## Abstract

Nowadays, radiotherapy has become an integral part of the treatment regimen in various malignancies for curative or palliative purposes. Ionizing radiation interacts with biological systems to produce free radicals, which attack various cellular components. Radioprotectors act as prophylactic agents that are administered to shield normal cells and tissues from the harmful effects of radiation. Melatonin has been shown to be both a direct free radical scavenger and an indirect antioxidant by stimulating antioxidant enzymes and suppressing prooxidative enzymes activity. In addition to its antioxidant property, there have also been reports implicating antiapoptotic function for melatonin in normal cells. Furthermore, through its antitumor and radiosensitizing properties, treatment with melatonin may prevent tumor progression. Therefore, addition of melatonin to radiation therapy could lower the damage inflicted to the normal tissue, leading to a more efficient tumor control by use of higher doses of irradiation during radiotherapy. Thus, it seems that, in the future, melatonin may improve the therapeutic gain in radiation oncology treatments.

## 1. Introduction

Radiotherapy is used for curative or palliative purpose in various patient malignancies [1]. It is estimated that about 50–70% of clinical oncology treatments are performed with either radiotherapy alone or a combination of radiotherapy and chemotherapy [2, 3]. However, radio sensitivity of normal tissues surrounding the tumor limits the therapeutic gain. The response of the normal tissues to therapeutic radiation exposure ranges from mild discomfort to life threatening, and the rates of such responses often depend on the amount and distribution of the radiation dose received by the tissue [4, 5].

Exposure of biological systems to ionizing irradiation leads to formation of free radicals including reactive oxygen species (ROS) as well as reactive nitrogen species (RNS) [6]. These agents impose damage to various biomacromolecules like the DNA, lipids, and proteins present in the cell [6].

The ability of certain radioprotective agents to provide protection against the damaging effects of ionizing radiation was first reported in 1949 [7]. The Walter Reed Research Institute of the United States Army synthesized and examined over 4,000 compounds in an attempt to find a suitable radioprotector in the late 1950s [8]. However, research efforts with synthetic radioprotectors in the past have led to little success primarily due to the various side effect problems [9]. The most efficient radioprotective agent of this type, which has been tested against lethal doses of X-rays and gamma irradiation in mice, is WR-2721, also called amifostine [8]. Although amifostine was reported as an applicable radioprotector in clinical radiation oncology, it was later found to cause some undesirable side effects such as hypotension, vomiting, nausea, sneezing, hot flashes, mild somnolence, and hypocalcaemia [10]. Results obtained from animal experiments show that antioxidant nutrients, like vitamin E [11] and a large number of plant productions [12], are protective against lethality and other radiation effects but to a lesser degree than most synthetic radioprotectors such as amifostine [11]. Since 1993, when melatonin (a pineal gland hormone) was first identified as a free radical scavenger [13], a large number of papers have been published confirming the ability of this radioprotective agent to shield against radiation-induced damage [5, 10].

In this review, from a radiobiological point of view, we present various features that make melatonin a potent agent in radiation oncology.

## 2. Melatonin: Synthesis and Distribution

Melatonin (N-acetyl-5-methoxytryptamine), which was discovered by A. Lerner in 1958 [7], is an endogenous compound synthesized by the pineal gland in the human brain. It has been reported that melatonin participates in the regulation of a number of physiological and pathological processes [10]. Initially, it was identified as a molecule related to neuroendocrine physiology, especially reproductive physiology [15]. Later on, melatonin was found to be involved in the control of circadian rhythms of diurnal species [15]. It is estimated that the half-life of melatonin in serum varies between 30 and 57 minutes [7]. Due to its small size and high lipophilicity, melatonin crosses the biological membranes and reaches all compartments of the cell [16]. Once synthesized in the pineal gland, melatonin is quickly released into the bloodstream [17, 18] and then into other body fluids [19], such as bile [20], cerebrospinal fluid (CSF) [21], saliva [22, 23], ovarian follicular fluid [24], and semen [25]. Also, it has been reported that melatonin can easily cross the blood-brain barrier and it is especially taken up* via* the choroid plexus [26, 27]. There has also been a report indicating that small amounts of unmetabolized melatonin are excreted in the urine [7]. Interestingly, it has been demonstrated that melatonin also gets produced in extrapineal organs and tissues such as the gastrointestinal tract, retina and lens, skin, immune and hematopoietic cells, some reproductive organs, and endocrine glands [28]. However, while pineal melatonin goes to the blood and CSF and can reach all tissues, retinal melatonin is thought to act only locally (i.e., within the eye) [5, 13, 29].

## 3. Antioxidative Effect of Melatonin

As mentioned earlier, melatonin has been shown to be a direct free radical scavenger and an indirect antioxidant* via* its stimulative effects on activities of antioxidant enzymes [5, 30] such as superoxide dismutase (SOD), glutathione peroxidase (GSH-Px), glutathione reductase (GR), and catalase (CAT) [15, 31–33]. Several studies have demonstrated that melatonin appears to ameliorate irradiation-induced injury in various organs including the spleen [34–36], liver [37–40], lung, colon, ileum [39], lens [29, 41], spinal cord [42–44], and brain [45] (Table 1).

It has been shown* in vitro* that melatonin directly scavenges ^•^OH, H_2_O_2_, and singlet oxygen (↑O_2_^•−^) and inhibits lipid peroxidation [15]. Melatonin also increases intracellular glutathione levels by stimulating the synthesis of the rate-limiting enzyme, *γ*-glutamylcysteine synthase, which inhibits the prooxidative enzymes nitric oxide synthase and lipoxygenase [15]. There is also some evidence that supports melatonin in stabilizing microsomal membranes and, thereby, probably helping them resist oxidative damage [50]. Moreover, melatonin has been shown to increase the efficiency of the electron transport chain to lower electron leakage and thus a reduction in generation of free radicals [51].

Koc and his coworkers have shown that melatonin acts as a radical scavenger in peripheral blood cells during whole-body irradiation in rats [46]. Results obtained from this study showed that both leukocyte and thrombocyte counts were significantly protected against 5 Gy gamma irradiation following a pretreatment with 5 mg/kg dose of melatonin [46]. Koc and colleagues also investigated the antioxidant role of melatonin (at 5 and 10 mg/kg) in the liver tissue against total-body gamma irradiation-induced oxidative damage with a single dose of 6 Gy [47]. The results demonstrated that in irradiated rats that were pretreated with melatonin (5 or 10 mg/kg) the liver tissue malondialdehyde (MDA) levels, as an end product of lipid peroxidation, were significantly lowered, whereas the SOD and GSH-Px activities were significantly increased. The authors concluded that pretreatment with melatonin may prevent irradiation-induced liver damage [47].

After exposure to 6 Gy whole-body irradiation, liver MDA and nitric oxide (NO) levels, two indicators of free radicals damage, were measured by Taysi et al. [37]. Gamma irradiation caused a significant increase in liver MDA and NO levels. Hepatic MDA and NO levels in irradiated rats that were pretreated with melatonin (5 or 10 mg/kg) were significantly decreased [37].

El-Missiry et al. [38] showed that treatment with 10 mg/kg melatonin for 4 days (daily) before acute irradiation (2 and 4 Gy) significantly abolished radiation-induced elevations in MDA and protein carbonyl levels (the oxidative stress markers) in the liver and significantly maintained hepatic glutathione content, glutathione-S-transferase (GST), and catalase (CAT) activities close to the control group values.

Radiation myelopathy (RM) is known as one of the most important complications in radiotherapy, and studies have exhibited dose- and time-dependent radiation effects [52–55]. Shirazi et al. [43] assessed the radioprotective effects of melatonin on biochemical, histopathological, and clinical manifestations of RM in the rat cervical spinal cord. Administration of melatonin markedly reduced MDA and increased GSH levels when compared with the control group.

Sharma and colleagues have shown that, due to its antioxidant properties, melatonin increases the immunity in squirrels, by protecting their hematopoietic system and lymphoid organs against 2.06 Gy X-ray-induced cellular toxicity [36]. In this study, total leukocyte and lymphocyte counts (TLC and LC) in the peripheral blood and lipid peroxidation (LPO) status, superoxide dismutase (SOD) activities, and total antioxidant status (TAS) were measured in the spleens of squirrels. Pretreatment with melatonin prior to the irradiation significantly increased LC, TLC, SOD activity, and TAS status compared to irradiation only groups, whereas LPO status was decreased [36]. In another study, a radioprotective effect of melatonin against 5 Gy gamma irradiation during the reproductively active and inactive phases (RAP and RIP) of Indian palm squirrels was evaluated. Results showed that melatonin treatment before irradiation significantly increased the LC and increased SOD activity in the spleen of squirrels compared with irradiation only group [35].

**Figure 1:**
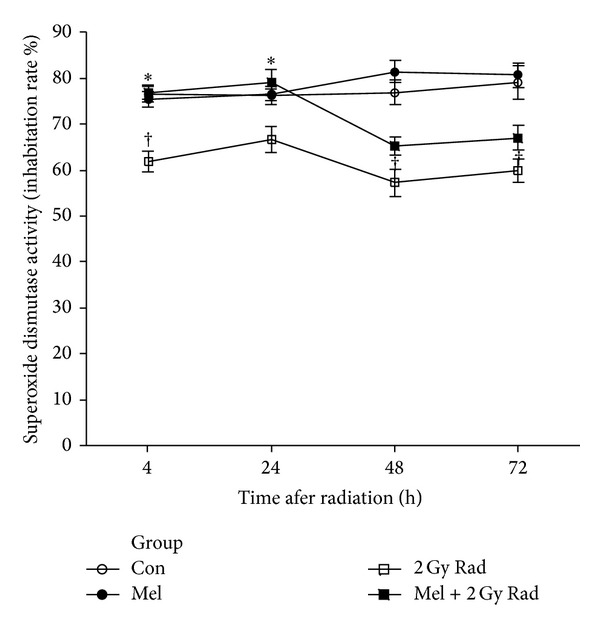
Effect of melatonin pretreatment (10 mg/kg) on SOD activity of serum at 4, 24, 48, and 72 hours after exposure to 2 Gy irradiation. Vertical bars represent mean + SEM, *n* = 5 for each group. Con: control; Mel: melatonin only; 2 Gy Rad: 2 Gy irradiation only; Mel + 2 Gy Rad: melatonin treatment and 2 Gy irradiation. ^†^ *P* < 0.05 when compared with their respective control groups, and *P < 0.05 when compared with their respective 2 Gy Rad groups [14].

**Figure 2:**
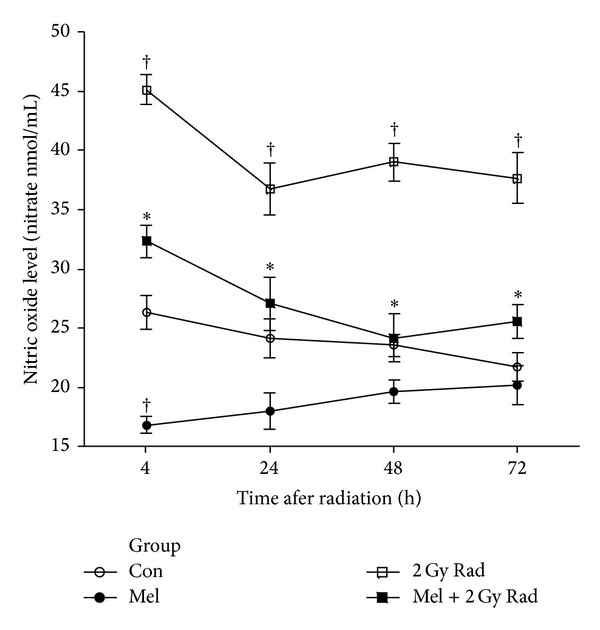
Effect of melatonin pretreatment (10 mg/kg) on NO levels of serum at 4, 24, 48, and 72 hours after exposure to 2 Gy irradiation. Vertical bars represent mean + SEM, *n* = 5 for each group. Con: control; Mel: melatonin only; 2 Gy Rad: 2 Gy irradiation only; Mel + 2 Gy Rad: melatonin treatment and 2 Gy irradiation. ^†^ *P* < 0.05 when compared with their respective control groups, and *P < 0.05 when compared with their respective 2 Gy Rad groups [14].

**Figure 3:**
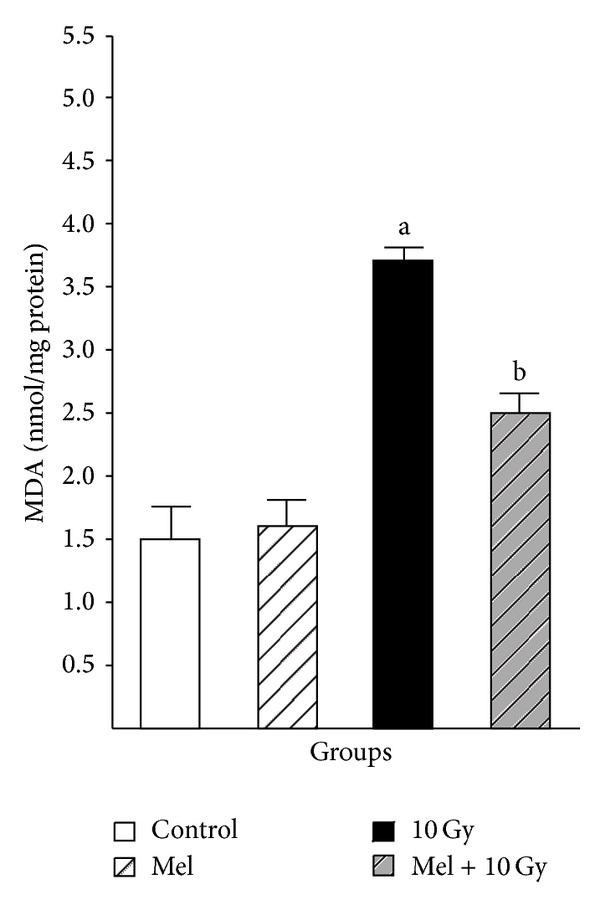
The effect of melatonin on MDA levels in rats' liver subjected to whole body gamma irradiation. Data represent mean ± standard error on the mean (SEM), *n* = 8 animals per group. ^a^ *P* < 0.05 compared to control group, and ^b^ *P* < 0.05 compared to the radiated groups [40].

**Figure 4:**
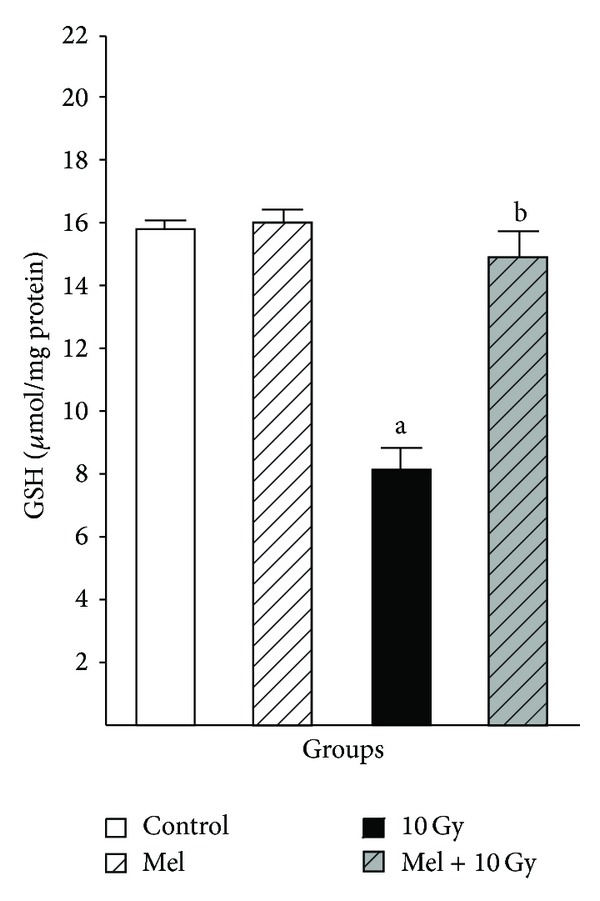
The effect of melatonin on GSH levels in rats' liver subjected to whole body gamma irradiation. Data represent mean ± standard error on the mean (SEM), *n* = 8 animals per group. ^a^ *P* < 0.05 compared to control group, and ^b^ *P* < 0.05 compared to the radiated groups [40].

In our recent study, we investigated the possible radioprotective effects of melatonin (10 mg/kg) against whole-body irradiation (2 and 8 Gy) induced oxidative damage on rats peripheral blood at different time points after exposure. Treatment with melatonin (10 mg/kg) ameliorates harmful effects of irradiation by increasing lymphocytes count (LC) as well as antioxidant enzymes activity and decreasing nitric oxide (NO) levels at all time points [14]. We concluded that 10 mg/kg melatonin is likely to be an adequate concentration for significant protection against lower dose of 2 Gy (Figures 1 and 2), while it does not offer significant protection against higher dose of 8 Gy. Therefore, it seems that radioprotective effects of melatonin are dose-dependent [14]. Moreover, our new data obtained from other studies showed that radiation exposure decreased levels of GSH and increased levels of MDA in the lens and liver (Figures 3 and 4) of rats, but these values were within normal limits when melatonin was administered [40, 41].

Overall, these findings support the antioxidant effects of melatonin against radiation-induced oxidative stress (Figure 7).

## 4. Antiapoptotic Effect of Melatonin in Normal Cells

Various* in vitro* and* in vivo* studies have presented evidence that radiation-induced apoptosis may be ameliorated by melatonin in rat neurons [56], retinal cells [57], and bone marrow cells [58], as well as thymocytes in mice [59]. Another study suggested that melatonin may modulate the apoptotic process in the small intestine of mice exposed to 2.5 Gy gamma irradiation [60].

Recently, we have shown that melatonin plays a role in reduction of radiation-induced apoptosis in rat's cervical spinal cord [62]. Results obtained from this study suggest that melatonin has protective effects against radiation-induced apoptosis. The principal finding in this work was that melatonin increased Bcl-2 gene expression versus a significant decrease in Bax gene expression in the irradiated spinal cord [62]. Therefore, one possible role of melatonin in prevention of spinal cord injury is to block radiation-induced apoptosis [62].

Sharma and colleagues have reported that melatonin, with its antiapoptotic properties, increased the immunity in the squirrels by protecting their lymphoid organs against 2.06 Gy X-ray-induced cellular toxicity [36]. In this study, apoptotic percentage on the basis of morphological changes and caspase-3 activity in melatonin pretreated group were decreased in the spleens of squirrels compared with the irradiation only group [36]. In another study by the same researchers, results demonstrated an inhibitory role of melatonin on caspase-3 activity in splenocytes during RAP and RIP following gamma radiation-induced caspase-mediated apoptosis [35]. These lines of evidence suggest that melatonin might have a role in reducing apoptosis by blocking caspase-3 activity [35].

**Figure 5:**
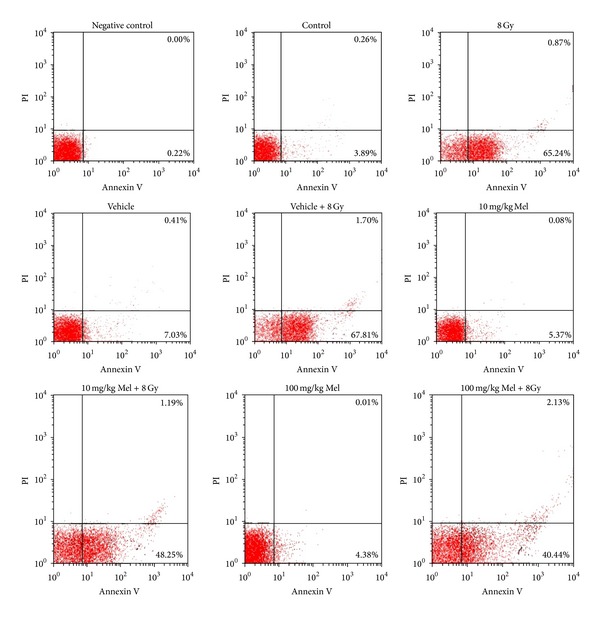
Effect of melatonin on radiation-induced apoptosis in rats peripheral blood lymphocytes. Rats were exposed to a single whole-body gamma ray dose of 8 Gy with or without melatonin (Mel) pretreatments (10 and 100 mg/kg IP 1 h before irradiation). Apoptotic and necrotic lymphocytes were analyzed by flow cytometric assay 4 h after irradiation. Representative dot plots of one set of three independent experiments of Annexin V and PI staining. Apoptotic lymphocytes (Annexin V^+^ and PI^−^) were displayed in the lower right quadrant and necrotic lymphocytes (Annexin V^+^ and PI^+^) were shown in the upper right quadrant [61].

**Figure 6:**
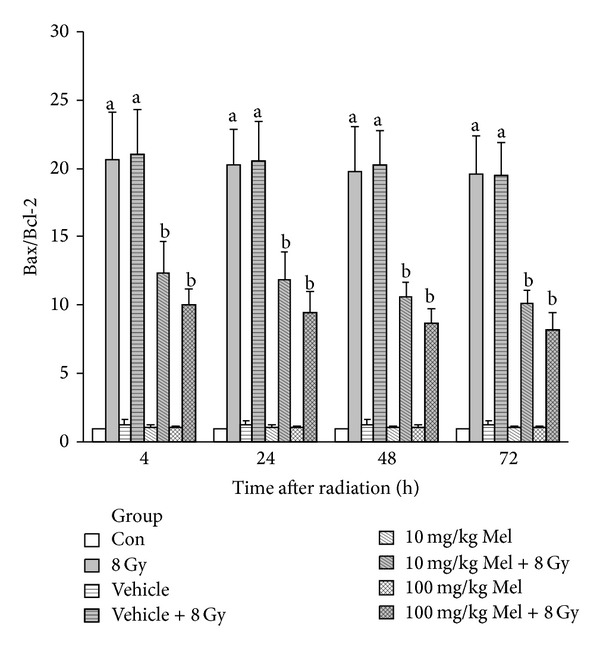
Real-time quantitative RT-PCR analysis of the fold change of bax/bcl-2 ratio at various time points after irradiation (relative to control). Values are expressed as mean ± SEM of three independent samples each performed in triplicate. ^a^ *P* < 0.01 compared to the control group, and ^b^ *P* < 0.01 compared to the 8 Gy and vehicle + 8 Gy groups [61].

In a study from our laboratory, we investigated the capability of melatonin (at 10 and 100 mg/kg dosages) in the modification of radiation-induced apoptosis and apoptosis-associated upstream regulators expression in rat peripheral blood lymphocytes [61]. Irradiation-only and vehicle-plus-irradiation groups showed a marked increase in the percentage of apoptotic lymphocytes, while melatonin pretreatments in a dose-dependent manner reduced it as compared with the irradiation-only and vehicle-plus-irradiation groups in all time points. This reduced apoptosis by melatonin was related to the downregulation of bax, upregulation of bcl-2, and therefore reduction of bax/bcl-2 ratio. Our results suggest that melatonin in these doses may provide modulation of bax and bcl-2 expression as well as bax/bcl-2 ratio to protect rat peripheral blood lymphocytes from gamma irradiation-induced apoptosis (Figures 5 and 6).

**Figure 7:**
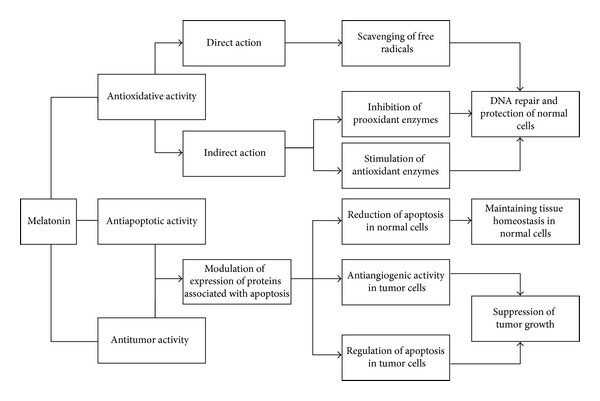
Possible mechanism for various effects of melatonin.

These studies document the role of melatonin in inhibiting radiation-induced apoptosis (Figure 7). However, our knowledge of melatonin as an antiapoptotic agent in healthy normal cells currently remains limited and needs further investigations.

## 5. Antitumor Effect of Melatonin

In addition to its antioxidant and antiapoptotic effects on normal cells, antitumor action of melatonin, including regulation of apoptosis or possible influence on angiogenesis of tumor (a major mechanism responsible for tumor growth and dissemination), has also been addressed [63]. Mills and colleagues in a systematic review of randomized controlled trials and meta-analysis observed a substantial reduction in the risk of death, low adverse events (or low side effects), and low cost of melatonin offer a great potential for melatonin in the treating of cancer [64].

In one of the initial studies, Lissoni and coworkers observed that, in patients with an advanced cancer of vascular endothelial growth factor (VEGF), melatonin may control the tumor growth, at least in part, by acting as a natural antiangiogenic molecule [65]. Cui and colleagues have reported a significant antiproliferative and apoptosis-inducing effects for melatonin in tumor cells by using western blot analyses for p53, Bax, and Bcl-2 expression in human umbilical vein endothelial cells [66]. All these effects were related to cell cycle arrest, upregulation of p53 and Bax, and downregulation of Bcl-2. The authors concluded that these results supported the antiangiogenic effect of melatonin in tumor cells [66]. Interestingly, oral administration of melatonin decreased the viability and volume of the tumor Ehrlich ascites carcinoma cells (EAC) implanted in female mice, delayed the progression of the cell cycle, and reduced the DNA content of these cells [67]. The depressed cell viability indicates that melatonin might be inducing apoptosis in EAC cells [67]. Moreover, the proliferating to apoptotic cell ratio in a colon cell line, induced by 1,2-dimethylhydrazine in mice, was significantly lowered when the animals were treated with melatonin [67].

Another group of researchers have examined the effect of melatonin administration on HepG2 human hepatocarcinoma cells [68]. Melatonin treatment induced apoptosis along with increased caspase-3 activity and poly (ADP-ribose) polymerase proteolysis. Proapoptotic effects of melatonin were related to cytosolic cytochrome C release, upregulation of Bax, induction of caspase-9 activity, and increased caspase-8 activity. The reduced cell proliferation and alterations of cell cycle were accompanied by a significant increase in p53 and p21 expressions. The authors concluded that, by inducing cell death and cell cycle arrest, melatonin might be useful as an adjuvant in hepatocarcinoma therapy [68].

Jang and colleagues have reported that as a radiosensitizer, melatonin enhances radiation-induced apoptosis in Jurkat leukemia cells, while it reduces radiation-induced apoptosis in normal mice splenocytes [69]. The reduced apoptosis by melatonin in normal cells was associated with the increase of Bcl-2 expression and a reduction of Bax/Bcl-2 ratio through a relative decrease of p53 mRNA and protein. The authors concluded that these differential effects on radiation-induced apoptosis by melatonin might involve regulation of p53 expression [69]. Thus, melatonin appears to have radioprotective effects for normal cells and it may also act as a radiation sensitizer for tumors in animal models [70].

According to the above lines of evidence, we propose some possible mechanisms for various effects of melatonin in Figure 7.

## 6. Melatonin and Other Radioprotectors

### 6.1. Melatonin and Amifostine

Among the various synthetic radioprotectors, amifostine has a large number of clinical applications and is currently in use as an adjuvant in radiotherapy [71, 72]. The synthesis of amifostine [S-2-(3-aminopropylamino) ethylphosphorothioic acid] was a major progress in the development of radioprotective drugs [73]. However, although amifostine was used well in many clinical trials, it remains to be expensive, and its use is limited to clinical settings because it must be given intravenously, and it has various undesirable side effects including nausea, vomiting, flushes, mild somnolence, hypocalcaemia, and hypotension [10, 74].

A significant advantage of amifostine over melatonin may be its differential and selective uptake only in normal but not in tumor cells [75, 76]. In contrast, melatonin, as a lipophilic molecule, can enter any cell compartment and does not seem to produce any serious side effects [77].

As mentioned earlier, some studies indicate that melatonin itself has antitumor effects [78]. Therefore, the combination of these agents might also prove useful because amifostine itself does not seem to reduce antitumor activity of another radioprotector [74]. Moreover, melatonin may also influence DNA repair enzymes directly and/or indirectly stimulate intracellular signals to activate genes responsible for enzymes involved in DNA repair [79]. In support of this hypothesis, the data reported by Kopjar and colleagues suggest that pretreatment with a combination of amifostine and melatonin prevents gamma irradiation-induced DNA damage in human peripheral blood lymphocytes* in vitro* [79]. Therefore, the authors have suggested that amifostine doses should be adjusted for optimal radioprotective effect in healthy normal cells, in order to inflict as few side effects as possible in cancer patients. Despite the limitations of using amifostine, this study has produced new insights that lead to the growing body of evidence that both amifostine and melatonin are effective radioprotectors [79]. However, before a combination of melatonin and amifostine can be used in clinical treatments, further experimental and clinical studies are needed for verification.

### 6.2. Melatonin and Vitamin E

Treatment with vitamin E, whether given prior to or immediately after irradiation, reduces radiation injury* via* its antioxidant effects by scavenging of free radicals [80]. Vitamin E also supports the immune system and protects bone marrow cells from the deleterious effects of gamma radiation by not only scavenging free radicals but also stimulating the DNA repair machinery [81]. Vitamin E has also been reported to maintain jejunal, ileal, and colonic fluid absorption in irradiated rats [82]. Interestingly, even when administered after 1 Gy of irradiation, vitamin E demonstrates protective effects against radiation-induced chromosomal aberrations (CA) and micronuclei (MN) in the mouse bone marrow [80].

Siu and colleagues compared the antioxidative capacity for vitamin E and melatonin and found a dose-dependent response to both, with melatonin being 7.2 times more potent than vitamin E [57]. This finding was further supported by works from Gitto et al., on rat liver homogenates [83], and Erol et al., on rat brain exposed to gamma irradiation [45]. Both melatonin and vitamin E protect cells from free radical attack by donating electrons to free radicals and neutralizing them. It should be noted that once vitamin E donates an electron, it becomes a radical itself, loses its antioxidant activity, and needs vitamin C to restore its antioxidant properties. By contrast, the antioxidant action of melatonin involves donation of two electrons; thus it does not turn into a free radical [34]. Hence, in contrast to other antioxidants such as vitamin E, the reaction products of melatonin with free radicals are themselves antioxidants [34].

Results obtained from a study by Yilmaz and colleagues show that melatonin may protect the bones from the damaging effects of radiation exposure, but there was no such protective effects observed for vitamin E [84]. In another study by Sharma and Haldar melatonin was found to be a more efficient antioxidant compared with vitamin E [34]. This might be due to a higher potency for melatonin in scavenging various free radicals generated after irradiation, as well as stimulating other antioxidative agents such as reduced glutathione (GSH) [34].

## 7. Dose- and Time-Dependent Treatment with Melatonin

Although the results outlined from the above studies (summarized in Table 1) suggest that melatonin may be effective for a variety of disorders, the optimal dose and mode of melatonin administration is not clear [67]. A wide dosage range for melatonin, from physiologic to pharmacologic concentrations, has been tested in different animal studies. The results of these studies indicate that both the acute and chronic toxicity of melatonin is extremely low [7].

Pretreatment with 0.1 mg/kg of melatonin, given orally for 15 consecutive days, affords potential protective effect against radiation-induced damage in mouse cerebellum [49]. It has been shown that melatonin, at a dose as high as 250 mg/kg, is nontoxic and that high doses of melatonin are effective in protecting mice from lethal effects of acute whole-body irradiation [10]. In human volunteers, oral administration of melatonin for a wide dosage range of 1–300 mg, and even up to 1 gram per day, for 30 days [85] resulted in no observable negative side effects [7]. In one of these studies by Vijayalaxmi and colleagues, a single oral dose of 300 mg of melatonin was given to four healthy, nonsmoking adult human volunteers. Peripheral blood samples were collected 5–10 min prior to and 1 and 2 hours after the ingestion of melatonin. The results obtained from this work indicated that the irradiated lymphocytes in the blood samples collected after melatonin ingestion exhibited a significant and time-related decrease in the extent of primary DNA damage (the length of DNA migration and fluorescence intensity in comet tail) and other genetic damages compared to similarly irradiated cells [86]. Vijayalaxmi et al. observed that protection with 10 mg/kg melatonin was significantly greater than 5 mg/kg melatonin against radiation-induced genetic damage in blood and bone marrow in CD2-F1 mice [87]. Melatonin, given in amounts of 0.1–10 mM, led to dose-dependent suppression of ROS produced by UV light [88]. Kim and Lee assessed the protective effects of melatonin (10 and 100 *μ*g) on the ovarian follicles at 2, 8, and 14 hours after exposure to 8.3 Gy of gamma irradiation. Results of this study suggest that radioprotective effects of melatonin related to its concentration [89]. In another important study on hepatocarcinoma cells, different doses (1000 and 10,000 *μ*M) of melatonin were administrated for 2, 4, 6, 8, and 10 days. Interestingly, the growth inhibition of hepatocarcinoma cells was both dose- and time-dependent and reached a maximum in cells treated for 10 days with 10,000 *μ*M [68]. According to these findings, it seems that higher concentration and/or long term of melatonin administration may produce more protection against deleterious effects of higher irradiation doses, which leads to a more efficient tumor control by use of higher doses of irradiation during radiotherapy.

Melatonin concentrations in the body are typically lower during the day and reach maximal levels at night in the darkness [7]. Ruifrok et al. reported a diurnal variation in apoptosis, with peak levels at 8:00 and minimal levels between 23:00 and 02:00 hours, in the small intestine of mice exposed to 2.5 Gy of gamma irradiation [60]. The physiological concentrations of radioimmunoassayable melatonin in the human blood are approximately 0–20 pg/mL during the day and 40–200 pg/mL during the night [90]. Therefore, administration of melatonin during evening in the darkness synergistically with great endogenously levels of produced melatonin in the body at this period may be more effective to protect against radiation-induced oxidative stress. Thus, in our opinion, it seems that radiation therapy with supplementary melatonin leads to more beneficial effects during the evening hours (in the darkness) and nighttime.

Moreover, in an aging study on rats, Reiter et al. observed much higher 8-OH-dG(8-hydroxy-2′-deoxyguanosine) as an index of DNA damage and MDA+4-HDA levels and increased microsomal membrane rigidity (indices of oxidative damage, which are also caused by ionizing irradiation) in a variety of organs collected from 25-month-old rats when compared with 2-month-old controls [91]. It is suggested that a reduction in physiological levels of melatonin may lead to the increase in oxidative damage in the elderly [15]. Therefore, based on these observations, it seems that higher concentrations and/or long term administration of melatonin must be used for aging patients than younger ones.

Intracellular melatonin does not exit from the cell. Instead, melatonin acts in the cell to protect it from oxidative/inflammatory damage. Additionally, the potent antioxidant and anti-inflammatory properties of melatonin depend on the high levels of the indoleamine that are found intracellularly. Thus, for protection against higher irradiation doses, high doses of melatonin are needed.

Despite the lack of clinical and experimental trials, according to these studies and our own observation, an optimal dose of melatonin may be achieved by a protocol such as (i) a low-dose pretreatment, preferably, in the evening, for example, one week or ten days prior to irradiation; (ii) a high-dose administration half an hour before exposure to irradiation or radiotherapy treatment; and (iii) a low-dose administration in the evening until the follow-up of patient after radiotherapy.

## 8. Conclusion

At the present, there is no truly ideal and safe synthetic radioprotector available. Thus, there have been numerous attempts directed at finding an effective radioprotective agent that can reduce and repair radiation-induced damage in healthy normal tissue. Although the mode and optimal dose of melatonin is still not clear, effective radioprotective actions show that including melatonin as an adjuvant in radiation therapy may decrease the normal tissue damage induced by irradiation, which leads to more tumor control by use of higher doses of irradiation during radiotherapy. Furthermore, due to its antitumor and radiosensitizing properties, treatment with melatonin is likely to increase damage to the tumor. Finally, based on the discussions presented earlier, we conclude that melatonin may be efficient in improving the therapeutic gain in future radiation oncology treatments. However, further experiments and clinical trials on this subject are still necessary to fully validate it.

## Figures and Tables

**Table 1:** Various studies presenting the protective effects of melatonin against irradiation-induced oxidative damage.

Tissue	Dose of irradiation	Dose of melatonin (b.w.)	Effect of melatonin on measured parameters in irradiated animals	Reference
Serum	2 and 4 Gy whole body single dose	10 mg/kg, daily for 4 days before irradiation	Albumin and total protein levels ↑, urea, total lipid, cholesterol levels and AST, ALP, and GGT activities ↓	[38]
Peripheral blood	5 Gy whole body single dose	5 mg/kg, 30 min before irradiation	Leukocyte and thrombocyte counts ↑	[46]
Peripheral blood	2 and 8 Gy whole body single dose	10 mg/kg, 30 min before irradiation	Lymphocyte count, SOD, GSH-Px, and CAT activity ↑, NO ↓	[14]
Liver, lung, colon, and ileum	8 Gy whole body single dose	10 mg/kg, immediately before and daily for 3 days after irradiation	MDA and MPO levels ↓, GSH level ↑	[39]
Liver	6 Gy whole body single dose	5 and 10 mg/kg, 30 min before irradiation	MDA level ↓, SOD and GSH-Px activity ↑	[47]
Liver	2 and 4 Gy whole body single dose	10 mg/kg, daily for 4 days before irradiation	Hepatic DNA and RNA contents, GSH level, GST and CAT activity ↑, TBARS and protein carbonyl levels ↓	[38]
Liver	10 Gy whole body single dose	30 mg/kg, immediately before and daily for 3 days after irradiation	MDA level ↓ and GSH level ↑	[40]
Cavernosum and urinary bladder	8 Gy whole body single dose	10 mg/kg, immediately before and daily for 3 days after irradiation	MDA level ↓ and GSH level ↑	[48]
Lens	5 Gy total cranium single dose	5 mg/kg, daily for 10 days before irradiation	MDA level ↓, SOD and GSH-Px activity ↑	[29]
Lens	5 and 8 Gy total cranium single dose	30 mg/kg, immediately before and 5 mg/kg daily for 10 days after irradiation	MDA level ↓ and GSH level ↑	[41]
Brain	7.2 Gy whole body in two equal fractions 12 h apart	100 mg/kg, daily for 5 days after irradiation	MDA level, rates of edema, necrosis and neuronal degeneration ↓	[45]
Cerebellum	4 Gy whole body single dose	0.01 mg/kg (orally), daily for 15 days before irradiation	GSH level ↑, TBARS level, number and volume of purkinje cells ↓	[49]
Spinal cord	22 Gy spinal cord area single dose	100 mg/kg, 30 min before irradiation	GSH level ↑, MDA level, demyelination and clinical sign of myelopathy ↓	[43]

*Note.* ↓: decrease; ↑: increase; MDA: malondialdehyde; MPO: myeloperoxidase; GSH: glutathione; SOD: super oxide dismutase; GSH-Px: glutathione peroxidase; AST: aspartate aminotransferase; ALP: alkaline phosphatase; GGT: gamma-glutamyltransferase; GST: glutathione-S-transferase; CAT: catalase; TBARS: thiobarbituric acid reactive substances; b.w.: body weight.
